# Crop and water productivity and profitability of broccoli (*Brassica oleracea* L. var. *italica*) under gravity drip irrigation with mulching condition in a humid sub-tropical climate

**DOI:** 10.1371/journal.pone.0265439

**Published:** 2022-03-17

**Authors:** Sanmay Kumar Patra, Ratneswar Poddar, Sanjit Pramanik, Ahmed Gaber, Akbar Hossain

**Affiliations:** 1 Department of Agricultural Chemistry and Soil Science, Bidhan Chandra Krishi Viswavidyalaya, West Bengal, India; 2 Department of Agronomy, Bidhan Chandra Krishi Viswavidyalaya, West Bengal, India; 3 Department of Soil and Water Conservation, Bidhan Chandra Krishi Viswavidyalaya, West Bengal, India; 4 Department of Biology, College of Science, Taif University, Taif, Saudi Arabia; 5 Department of Agronomy, Bangladesh Wheat and Maize Research Institute, Dinajpur, Bangladesh; Soil and Water Resources Institute ELGO-DIMITRA, GREECE

## Abstract

Strategic irrigation planning and soil water conservation measure can be rewarding for increasing agricultural productivity in a humid subtropical climatic environment. Field experiments were conducted to evaluate broccoli yield response to crop water productivity (CWP), irrigation water productivity (IWP) and economics under varied irrigation regimes and mulching. Four levels of irrigation: surface irrigation at 1.0 IW/CPE (irrigation water to cumulative pan evaporation, I_1_), drip irrigation at 0.6 (I_2_), 0.8 (I_3_) and 1.0 (I_4_) of crop evapotranspiration (ETc) and three mulches: none (M_0_), black polythene mulch (BPM, M_1_) and paddy straw mulch (PSM, M_2_) were examined. Among these treatments I_3_M_1_ produced the higher yield (19.17 t ha^-1^), CWP, IWP and maximum benefit-cost ratio (BCR), being almost competitive with I_3_M_1_. Under scarcity of water, I_2_M_1_ was an alternative. Drip irrigation could save 21.2–52.7% water over surface irrigation which accommodated 17.1 to 53.3% additional area under irrigation. Yield response factor and water-yield production function suggested the potential yield decrease in relation to increased deficit irrigation. However, a deficit drip irrigation scheduling with 0.8 ETc at a 3-day interval is optimum for increased curd yield, water productivity and economics of broccoli.

## 1. Introduction

Broccoli (*Brassica oleracea* L. var. *italica*) is an important dark green coloured winter vegetable crop grown extensively in the temperate, tropical and sub-tropical regions of the world. It belongs to the Brassicaceae family in the member of cole group. It is palatable with fairly rich sources of nutrition containing dietary fiber (2.6%), protein (3.3%), fat (3.3%), carbohydrate (5.5%), vitamin A, B and C, antioxidants, phytochemicals and minerals like P, Ca, Mg, Mn, Fe, Zn and Se [1, 2].

The broccoli crop is shallow-rooted and sensitive to water stress. It requires a frequent and light supply of irrigation and chemical fertilization to avoid water and nutrient deficiencies for attaining maximum production [3]. The optimum availability of water and nutrients in soil is the determining factor that preferably influences the growth, yield and quality of produce. The abundant or limited supply of these resource inputs in any stage of the growth cycle deteriorated crop growth and productivity [4].

Water is one of the most critical environmental factors affecting the sustainable production of crops. Inadequate irrigation causes water stress and reduces the photosynthesis, metabolic processes and nutrient uptake in plant resulting in a decrease in cell division and cell elongation and hence reduced growth and yield [5]. Moderate to severe water stress significantly decreases photosynthesis, while mild stress has less effect on photosynthesis. In deficit irrigation management both amount and timing of water stress are important because some crop growing stages are more sensitive to water shortage than others. In some cases, the crop is tolerant to a certain level of water stress either during a particular period or throughout the total growing period, but after the limit, it starts losses in growth and yield [6]. The excessive irrigation, on the other hand, rendered losses of water and nutrients in deep percolation below the root zone and depressed yield even led to groundwater pollution. Under conditions of limited water supply, controlled irrigation scheduling is considered a critical deficit irrigation management strategy for minimizing water deficits during the most sensitive growing periods and ensuring a favourable soil moisture regime in the root zone by applying a precise amount of water which helped increase the quantity and quality of products with higher water use efficiency and profitability [7, 8]. Different meteorological based irrigation scheduling approaches could save considerable amounts of water and energy, besides thwarting soil water deficit below the threshold level for specific soil, crop and climatic conditions [1]. Therefore, in the prevalent agro-climatic region, the strategic development of irrigation scheduling by applying the exact amount of water to replenish the soil moisture to the desired level is necessary for the full exploitation of scarce water resources. The conventional surface method of irrigation which is widely practiced results in inefficient use of irrigation water due to losses in evaporation, deep percolation and distribution [9]. However, micro-irrigation particularly drip irrigation is the most efficient and economically viable modern irrigation method and proved its superiority over other conventional methods of irrigation because of its unique ability to apply light but frequent water directly in the proximity of the root zone of each plant [10]. It also provides daily requirements of water and maintains a high soil matric potential in the rhizosphere to reduce plant water stress [11, 12]. This hi-tech irrigation system with proper irrigation scheduling improved crop yield and quality, increased water use efficiency by reducing runoff and evaporation, and safeguarded the ground-water contamination due to less deep percolation [13]. Drip irrigation is preferred for vegetable and fruit crops having high commercial value; however, it can be profitable for high and medium water requiring different cereal and oilseed crops such as rice, wheat, maize, corn, sunflower, soybean and groundnut and fibre crop like cotton as less irrigation water is available for production purposes due to other forms of anthropogenic water consumption, ecological use and above all, climate change [14–16]. In the water-scarce region, a low-cost gravity drip irrigation system provided with user-friendly accessories is considered techno-economically feasible and viable for small-sized farms. Kashyap et al. [17] reported that drip irrigation treatments irrespective of mulching showed significantly better performance in plant height, leaf number and other growth parameters and curd yield in broccoli and the water use efficiency was highest in drip irrigation at 60% evapotranspiration with 74.3% water saving. Haris et al. [12] found that irrigation at 80% evapotranspiration registered significantly higher crop yield and daily irrigation gave significantly higher yield than once two days and three days schedules. In a similar study, Patle et al. [18] observed the highest yield and water use efficiency of broccoli with gravity drip irrigation at 80% evapotranspiration along with mulch in per humid ecoregion.

Several empirical modeling approaches can be used as a powerful tool to evaluate crop production under variable water supply conditions. This will provide a convenient means to predict crop yield in response to irrigation application concerning the water management. The crop water productivity or, water use efficiency denoting the relationship between marketable yield and the seasonal water use by the plant through evapotranspiration is an important indicator to express the resource use efficiency and can provide an assessment of crop performance under different irrigation strategies [9, 19]. The water-yield production function under limited water resource conditions is computed by regression analysis using data on seasonal irrigation water applied and yield obtained. The relationship between irrigation water versus yield may be linear, curvilinear or any other configuration depending upon the irrigation management [20, 21]. The linear relationship indicates the increase in yield proportional to the increasing water application and the amount of water is fully used for crop production [20]. On the other hand, the curvilinear relationship points out that the crop does not exploit the full amount of water applied for yield increase; rather a significant portion of water is wasted in deep percolation [22]. This function is also important for prioritizing the allocation of optimum irrigation water for the desired yield and will be useful for researchers and policymakers for designing improved and efficient water management plans in different climatic conditions for obtaining potential benefits [9, 23]. The crop water production function is also expressed by the crop yield response factor (Ky) which links relative yield decrease to relative evapotranspiration deficit. Such quantitative expression integrates the weather, crop and soil conditions that make crop yield less than its potential yield in response to deficit evapotranspiration [24]. In limited irrigation water, but distributed uniformly over the total growing season, crops with higher Ky values will suffer a greater yield loss than the crops with lower Ky values. It is a deficit irrigation management strategy for production planning where the possible losses in crop yield with a reduction in the water supply can be quantified and the adjustments required in water supply being made for minimizing such losses [25]. The understanding of Ky makes it possible to select the best crops and cultivars for a specific site, soil and climate according to the quantum of water deficit condition and concurrent yield losses.

Mulching is an effective agronomic tool that has been advocated for conserving soil moisture, preventing weed, pest and disease infestation, moderating the physical, chemical and biological environment of soil, promoting hydrothermal regimes, minimizing water and nutrient losses, improving the nutrient recycling and organic matter build-up and increasing the yield and water use efficiency of crops [26]. Different organic materials like crop residues, leaf debris, husk, grass, hyacinth and inorganic polyethene sheet are used as mulches. The adverse effect of deficit irrigation on crop yield could be healed by adopting proper moisture conservation methods like the use of mulches [27]. Plastic mulches are now widely used on many winter season vegetable crops which substantially reduce soil water evaporation, curtail the irrigation requirement and increase the water use efficiency when used efficiently in combination with drip irrigation systems [28, 29]. Higher growth and yield of broccoli in water hyacinth mulch followed by paddy straw mulch as compared with black polythene mulch was obtained by Islam et al. [5]. In limited water availability areas, the beneficial role of black polythene mulch in stimulating soil water and nutrient uptake for higher crop growth and yield through their influence on soil moisture conservation and temperature regulation is well known [30, 31].

In recent years commercial cultivation of the newly emerging vegetable crop broccoli is gaining popularity among the farmers due to its richer nutritional value, high productivity and good marketable price and higher profit margin. The crop is grown during the winter season when there is the occurrence of low rainfall with high evaporation in the atmosphere. The crop requires a high volume of irrigation water to meet the evapotranspiration demand. Most of the farmers usually follow the conventional furrow method for irrigating the crop which is quite inefficient and renders excessive losses of water and nutrients in deep percolation below the root zone. Besides, the crop experiences adverse effects of cyclic over-irrigation and water stress in several growth stages resulting in poor water and nutrient use efficiencies, depressed crop yield and sub-standard quality of the produce. In consideration of the above circumstances, the objectives of the present study were to explore the effects of gravity drip irrigation schedules and mulch materials on curd yield, crop water and irrigation water productivity and economics; to develop and examine the water-yield production model; and to determine the yield response to water stress for broccoli in the lower Indo-Gangetic plains of a humid subtropical climate of eastern India.

## 2. Materials and methods

### 2.1. Description of experimental site, climate and soil

The field experiment was conducted at the Central Research Farm, Bidhan Chandra Krishi Viswavidyalaya, Gayeshpur, Nadia, West Bengal for three consecutive winter/*Rabi* seasons (November-January) of 2014–15, 2015–16 and 2016–17. The area belongs to the Indo-Gangetic plains region under the humid subtropical climate of eastern India. The experimental site lies at 22^0^58’31’’ N latitude and 88^0^26’20’’ E longitude with an altitude of 9.75 m above AMSL. The weather is hot and humid during the summer season (May-June) but dry and cold during the winter season (December-January). December and January are the coldest months of the year, while May and June are the hottest months of the year. The mean monthly temperature ranges from 8.5 to 23.7 ^0^C in winter and 25.4 to 37.6°C in summer. Sporadic rain during April-May and November-February is the common feature of the region. Monsoon ceases in October and the cold season sets in November. The soil of the experimental site is sandy loam in texture and classified as TypicFluvaquept with good drainage and water transmission characteristics. The physical and hydro-physical properties along the depth of the soil profile are furnished in Table 1.

**Table 1:** Physical and hydro-physical properties of the experimental soil.

Soil depth (cm)	Soil texture (%)			BD (Mg m^-3^)	HS (cm hr^-1^)	Infiltration (cm hr^-1^)	FC (%, w/w)	PWP (%, v/v)	AWC (%, v/v)
	Sand	Silt	Clay						
0–15	70.17	15.75	14.08	1.49	53.25	16.03	55.83	16.03	55.83
15–30	72.41	16.24	11.35	1.53	50.37	14.81	53.34	14.81	53.34
30–45	78.92	12.27	8.81	1.58	48.78	13.40	53.07	13.40	53.07
45–60	74.56	14.01	11.36	1.47	45.38	13.18	48.30	13.18	48.30

^a^BD = bulk density, HS = hydraulic conductivity, FC = field capacity, PWP = permanent wilting point, AWC = available water content.

**Fig 1:**
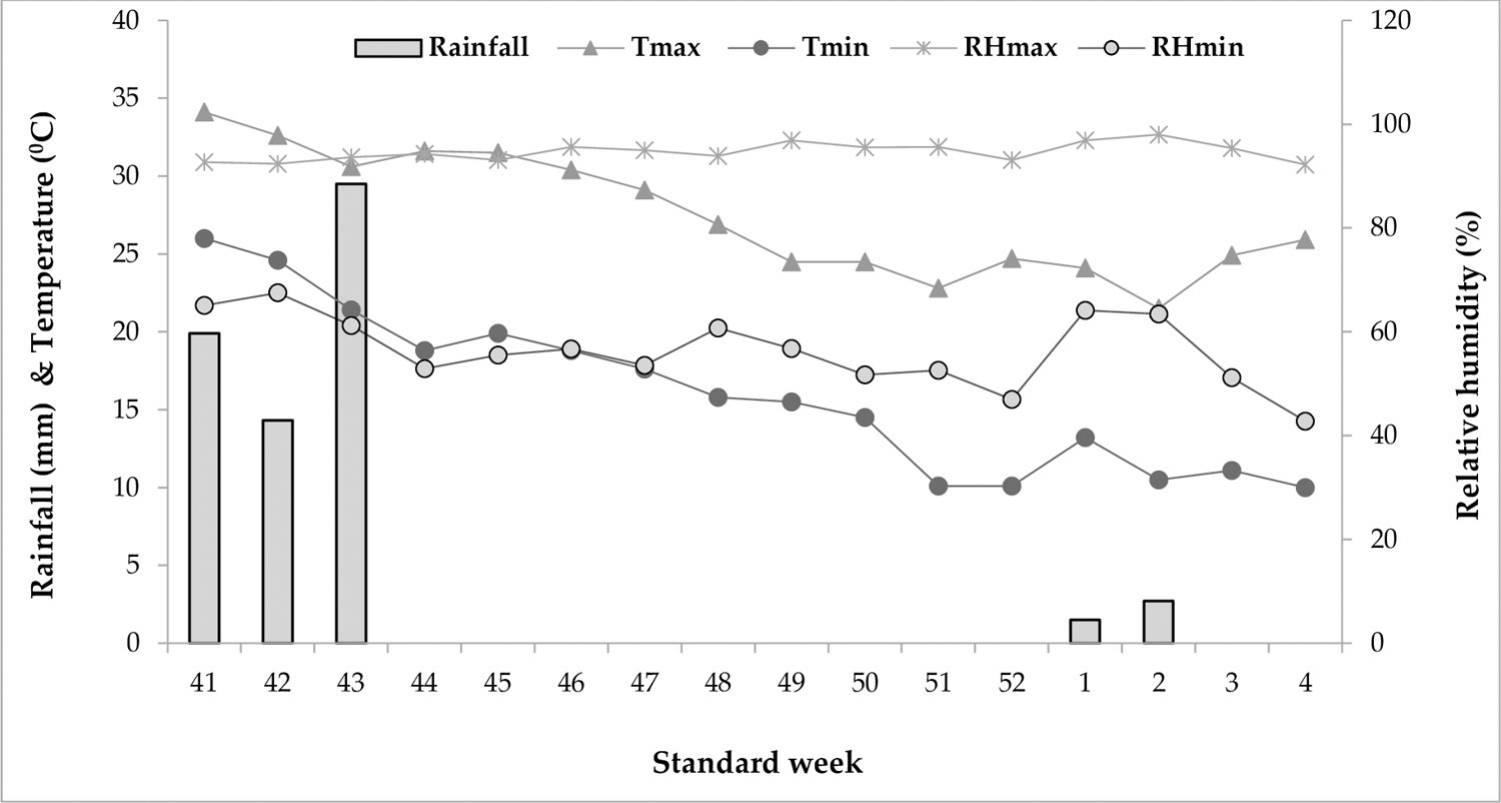
Climatological parameters during the cropping period (average weekly data of 3-year).

The soil had pH 6.83, EC 0.38 dSm^-1^ and organic carbon 5.8 g kg^-1^ with available N, P and K contents were 183.7, 31.5 and 180.4 kg ha^-1^, respectively. The mean weekly meteorological parameters averaging over the three years during the experimental period is furnished in Fig 1.

The total mean rainfall received during the growing period was 19.85 mm. The pan evaporation loss was in the range of 0.91 to 1.98 mm day^-1^ from November to January. The depth of the ground-water table varied from 3.8 to 4.7 m bgl.

### 2.2. Experimental treatments and design

The treatments consisted of four levels of irrigation *viz*., surface irrigation at 1.0 irrigation water to cumulative pan evaporation (IW/CPE) at 50 mm, and drip irrigation at 60, 80 and 100% of crop evapotranspiration (ETc) assigned in main plots and three mulch conditions *viz*., no-mulch, black plastic mulch (BPM) and paddy straw mulch (PSM) in sub-plots were laid out in a split-plot design with twelve treatments and three replications.

### 2.3. Agronomic interventions

Broccoli (*cv*. Green magic) seeds were sown on 13^th^ October every year in the nursery bed at a depth of 5 cm with a spacing of 10 cm between the rows. Healthy and disease free 30-day old seedlings were transplanted in the field on 11^th^ November of each experimental year with crop geometry of 45 cm row to row and 45 cm plant to plant distance in a net plot size of 4 × 3 m^2^. Just after planting, common irrigation at 20 mm depth was given to all plots for uniform plant establishment and maintaining an identical irrigation regime. The crop was harvested on 30 January in each year and curd yield for each treatment and replication was recorded. Just before planting, BPM of 25 μ thickness having small holes of 60 mm diameter at a distance of 45 cm was spread over the prepared field and seedlings were transplanted in the holes. PSM at 5 t ha^-1^ on an air-dry basis was also placed uniformly over the plot at 4–5 cm thickness. The routine standard agronomic and plant protection measures were adopted equally in all the treatments.

## 2.4. Soil fertilization

All treatments received recommended doses of 100, 60 and 40 kg ha^-1^ of N, P_2_O_5_ and K_2_O in the form of urea, single superphosphate and muriate of potash, respectively. Half N and full P_2_O_5_ and K_2_O were applied as a basal dressing at the time of planting and the remaining half dose of N was top-dressed at 45 days after transplanting. Farmyard manure at 15 t ha^-1^ was incorporated in all plots before transplanting and thoroughly mixed up with the soil.

### 2.5. Irrigation schedules

Irrigation treatments were given based on surface irrigation at 1.0 IW/CPE with 50 mm depth at 19–22 day intervals whereas gravity-fed drip irrigation was scheduled at 60, 80 and 100% of evapotranspiration (ETc) at the 3-day interval. The number of irrigation required for surface and drip irrigation systems was 3 and 16, respectively. The irrigation water was lifted to fill a 500-litre capacity overhead tank placed at 3.2 m height from local ground level to facilitate water movement by gravitational force. Drip lateral lines of 12 mm diameter were laid in between the two rows. Two drippers per plant were provided on either side of plants at 45 cm apart below the plastic and paddy straw mulch treatments. The dripper discharge was 1.8 lph with a pressure of 0.48 kg cm^-2^. The discharge rate of drippers was taken to adjust time of operating the drippers according to the volume of water precisely required based on prescribed irrigation schedules throughout the growing period. Running time (RT) for drip irrigation was calculated as,

RT = volume of water applied divided by number dripper × dripper discharge rate.

The quantity of water applied in surface irrigated treatments was measured with a Parshall flume placed at the entry point of each sub-plot. The irrigation was stopped at 10 days before harvesting of broccoli.

### 2.6. Computation of irrigation water requirement

The amount of irrigation water applied to broccoli crop by gravity-fed drip irrigation system was calculated by using the equation given below:

I=Ep×Kp×Kc×Wp (1)

where, I is the irrigation water applied (mm),Ep is the pan evaporation (mm), Kp is the pan factor, K_c_ is the crop coefficient and Wp is the percentage of wetted area. The evaporation data were obtained from a USDA Weather Bureau Class A Pan located inside the experimental site on wooden support at a height of 15 cm above the soil surface and readings were recorded daily. The K_p_ value was taken as 0.7. The K_c_ value adopted for early growth, midseason, button formation and head development stage was 0.70, 0.88, 1.05 and 0.95, respectively [32]. The W_p_ value was taken as 100% assuming that lateral interval is equal to the spaces between drippers. The performance of the gravity drip system was excellent as the emission uniformity of the system was more than 90%.

### 2.7. Estimation of actual crop evapotranspiration

The seasonal water use or actual crop evapotranspiration (ETa) for broccoli during the entire cropping period was computed using the field water balance equation [33] as

$${ET}_{a}=I+P-D-R\pm \Delta SW$$ (2)

where, I is the irrigation water (mm), P is the precipitation (mm), D is the drainage (mm), R is runoff (mm) and ΔSW is the change in soil water storage in 0–60 cm profile between planting and harvest time (mm). The portion of precipitation retained in the active root zone depth which was used to meet the crop evaporative demand was considered effective rainfall (R_e_) and was calculated as, R_e_ = P—D.

Runoff was assumed zero as all the sub-plots were surrounded by 50 cm wide bund and irrigation water applied was carefully managed to prevent overflow.


$$Thus,\;{ET}_{a}=I+R_{e}\pm \Delta SW$$ (3)

**Fig 2:**
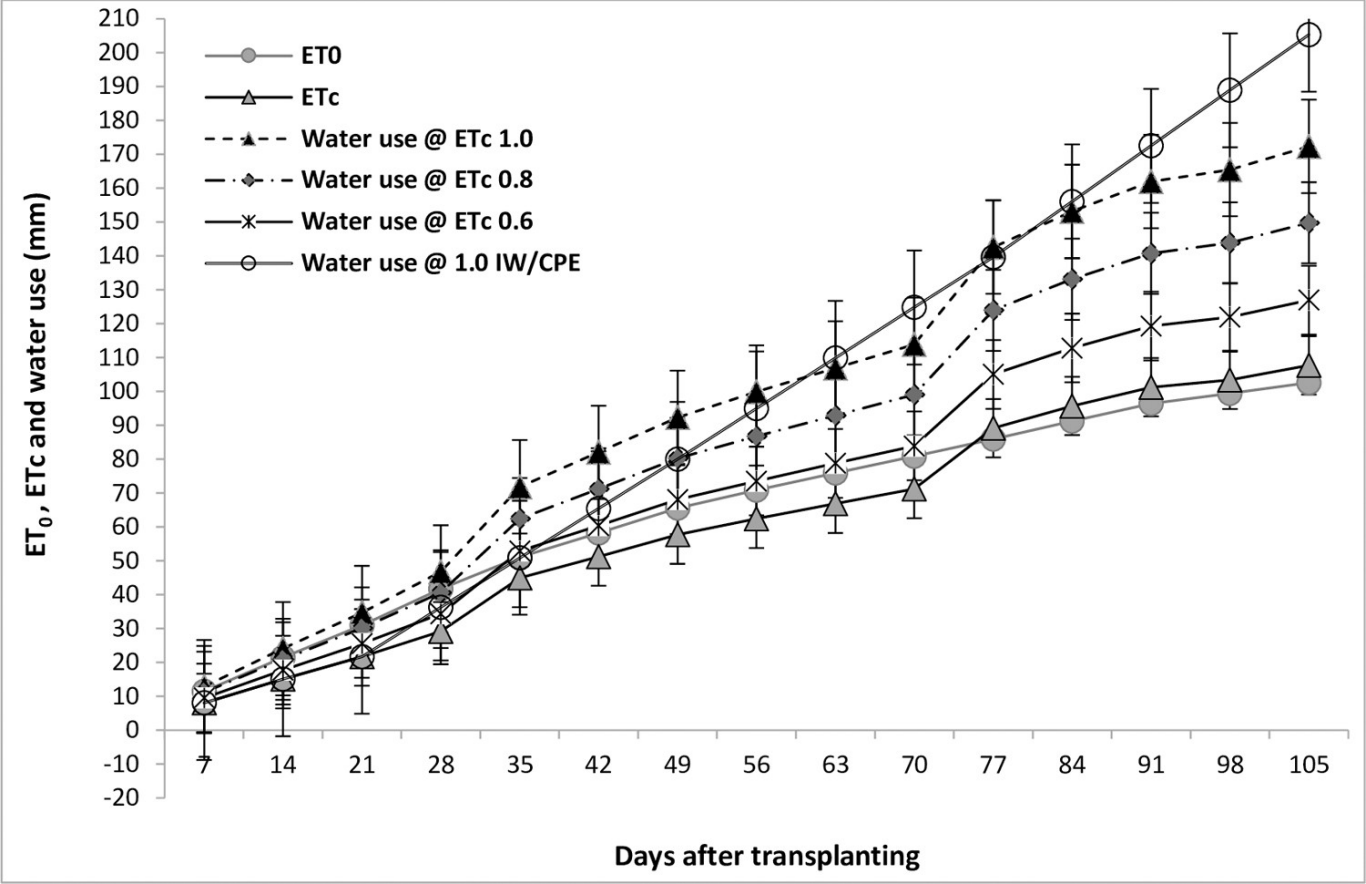
Weekly sum of reference evapotranspiration (ET_0_), crop evapotranspiration (ETc) and crop water use at different irrigation schedules during the cropping period (average weekly data of 3-year).

In the current research, the weekly sum of reference evapotranspiration (ET_0_), crop evapotranspiration (ETc) and crop water use at different irrigation schedules during the cropping period (average weekly data of 3-year) are presented in Fig 2.

### 2.8. Evaluation of water productivity

The crop water productivity (CWP) is the ratio of curd yield obtained for each treatment to the seasonal amount of water depleted by the crop in the process of evapotranspiration and was calculated by the following relationship [19]:

CWP(kgm−3)=YETa (4)

where, Y = fresh curd yield (kg ha^-1^), ETa = actual crop evapotranspiration (m^3^ ha^-1^).

The irrigation water productivity (IWP) expressed as kg m^-3^ was estimated as the ratio of curd yield (Y) in kg ha^-1^ to the amount of irrigation water applied (IW) in m^3^ ha^-1^ [34] *i*.*e*.


$$IWM=\frac{Y}{IW}$$ (5)

### 2.9. Computation of additional cultivated area

The area that could be cultivated by using the irrigation water saved from the imposition of drip irrigation is the additional cultivated area (X) which is calculated by the equation given below:

$$X=\frac{Water\;saved\;from\;drip\;irrigation}{Total\;water\;used\;for\;drip\;irrigation}\times 100$$ (6)

### 2.10. Water-yield production function

**Fig 3:**
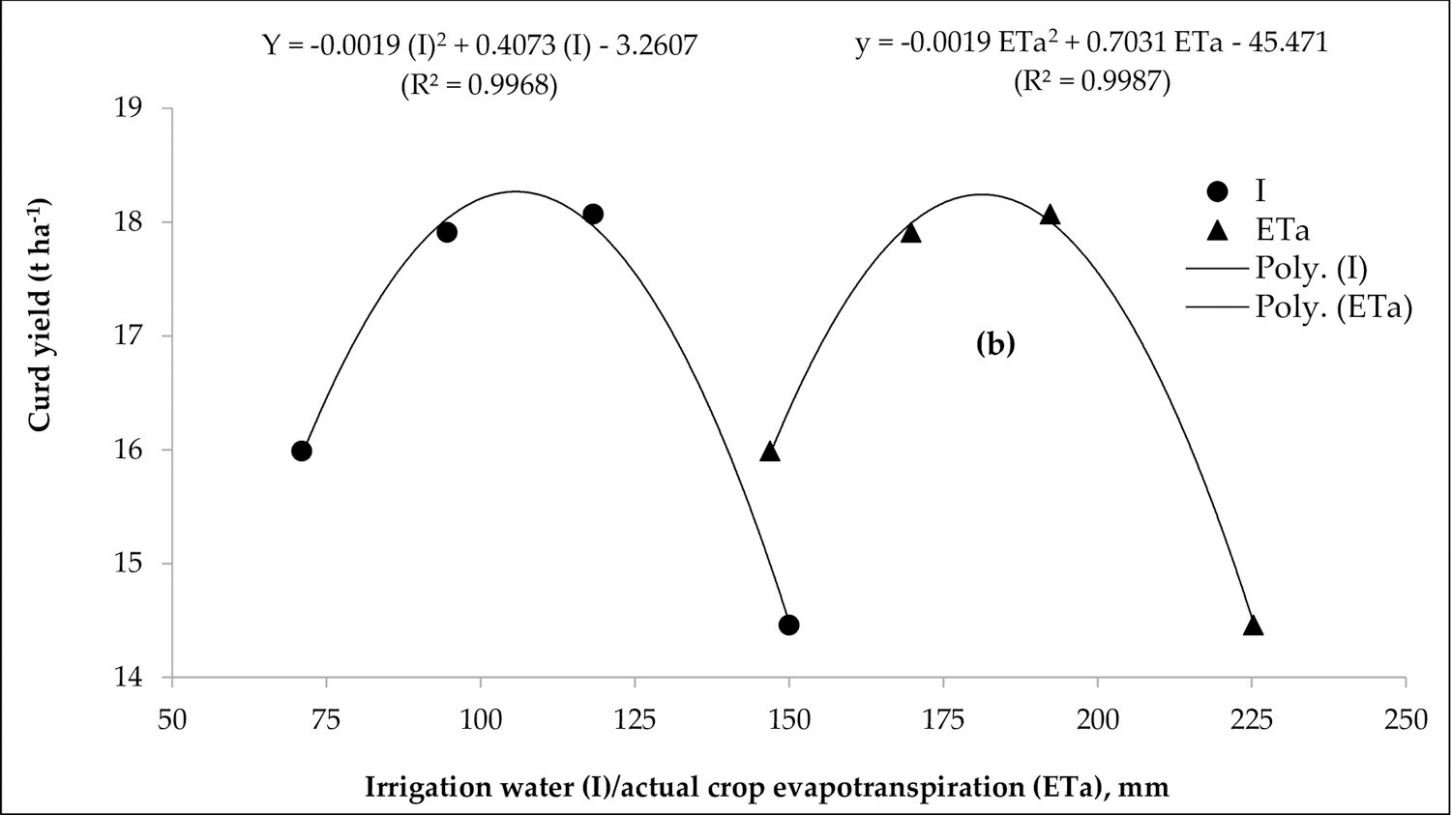
Relationship between (a) irrigation water (I) vs. curd yield and (b) actual crop evapotranspiration (ETa) vs. curd yield for broccoli for different levels and methods of irrigation (average data of 3-year).

The relationships between yield and irrigation water applied (I) through surface irrigation and drip irrigation treatments as well as actual crop evapotranspiration (ETa) were determined by non-linear regression analysis. In this model approach, all the water balance components including the entire irrigation treatments under drip irrigation and surface irrigation were taken into consideration for projecting the curd yield. Broccoli yield (y) was taken as a dependent variable and plotted against I and ETa as independent variables to derive mathematical water-yield production functions (Fig 3A and 3B).

### 2.11. Crop yield response factor

The overall response of broccoli curd yield to deficit irrigation during the growing season was quantified through the yield response factor (K_y_) and was computed using Stewart’s model [24]

$$K_{y}=\frac{1-Ya/Ym}{1-ETa/ETm}$$ (7)

where, Ya and ETa are the actual curd yield (t ha^-1^) and actual evapotranspiration (mm), respectively for the deficit irrigation treatments (I_1_ and I_2_); Y_m_ and ET_m_ are the maximum curd yield (t ha^-1^) and maximum evapotranspiration (mm) for full irrigation treatment I_3_, respectively and 1-Ya/Ym is the relative decrease in curd yield to the corresponding relative deficit in evapotranspiration (1-ETa/ETm).

### 2.12. Economics

The economic feasibility of the drip irrigation system was worked out through benefit: cost ratio analysis. The seasonal cost of the gravity drip system with a 4 HP water pump included 4% depreciation, 10% interest rate and 2% for repair and maintenance calculated from the fixed cost. The life span of the gravity drip irrigation system was considered to be 10 years. The cost of cultivation accommodated expenses incurred in main field preparation, sowing and transplanting, intercultural operation, seed, fertilizer, crop protection measures, irrigation water, crop harvesting and processing including application charges. The gross return was worked out by accounting for the prevailing average market price of the product during the experimental period. The net return was estimated by deducting the total cost of cultivation from the gross return. The benefit-cost ratio (BCR) of each treatment was calculated by dividing the net return by the cost of cultivation.

### 2.13. Statistical analysis

The year-wise curd yield data obtained for different treatments imposed were subjected to analysis of variance using software packages of MS Excel and SPSS 16.0 version. Statistical significance between means of individual treatments was assessed using the least significant difference (LSD) test at *P*≤0.05 [35]. Since the variation in data across the experimental years was estimated to be homogeneous by performing Bartlett’s chi-square test, and interactions between irrigation and mulching were almost similar and therefore the year variance was pooled with the experimental error variance to draw the inferences.

## 3. Results

### 3.1. Effect of irrigation and mulch on curd yield

The curd yields of broccoli were significantly influenced by different levels of drip irrigation over the surface irrigation in each experimental year (Table 2).

**Table 2:** Effects of different methods and levels of irrigation and mulches on broccoli curd yield.

Treatment	Curd yield (t ha^-1^)			
	2014–15	2015–16	2016–17	Pooled
Irrigation (I)				
I_1_	15.31	13.57	14.50	14.46
I_2_	16.67	15.47	15.83	15.99
I_3_	17.76	17.62	18.35	17.91
I_4_	17.87	17.73	18.61	18.07
SEm±	0.11	0.12	0.09	0.08
LSD (*P*≤0.05)	0.39	0.43	0.32	0.29
Mulching (M)				
M_0_	15.42	15.02	15.56	15.33
M_1_	18.43	16.88	18.41	17.91
M_2_	16.86	16.39	16.50	16.58
SEm±	0.14	0.17	0.14	0.12
LSD (*P≤* 0.05)	0.44	0.51	0.42	0.38

I_1_: surface irrigation at IW/CPE 1.0, I_2_: drip irrigation at 0.6 ETc_,_ I_3_: drip irrigation at 0.8 ETc, I_4_: drip irrigation at 1.0 ETc; M_0_: nomulch, M_1_: black plastic mulch, M_2_: paddy straw mulch, ETc: crop evapotranspiration.

The imposition of the incremental level of drip irrigation from 0.6 ETc up to 1.0 ETc consistently increased the yield at varying extents in all the years. The analysis of 3-year pooled data revealed that optimal drip irrigation at 1.0 ETc recorded the highest curd yield of 18.07 t ha^-1^ which was statistically at par with moderate deficit drip irrigation at ETc 0.8 (17.91 t ha^-1^), but superior to the higher deficit drip irrigation at ETc 0.6 (15.99 t ha^-1^). Surface irrigation at 1.0 IW/CPE recorded significantly the lowest yield (14.46 t ha^-1^). On an average, drip irrigation at 1.0, 0.8 and 0.6 of ETc increased the curd yield by 25.0, 23.9 and 10.6%, respectively over the surface irrigation.

Mulch treatment irrespective of methods and level of irrigation had a significant effect on curd yield over without mulched treatment in all the years and in their pooled data. However, BPM significantly produced a mean maximum yield (17.91 t ha^-1^) over that of PSM (16.58 t ha^-1^). The overall increase in curd yield was 16.83% for BPM and 8.15% for PSM in comparison with the no-mulch condition.

The interaction effects between irrigation methods and mulches on yield were significant in all the experimental years (Table 3). Averaging over the years, drip irrigation at 1.0 ETc complemented with BPM significantly recorded maximum curd yield (19.34 t ha^-1^) which was on par with drip irrigation at ETc 0.8 coupling with BPM (19.17 t ha^-1^). The addition of mulch in both methods of irrigation resulted in higher curd yield than in no-mulch treatment and that too in BPM than in PSM. Surface irrigation without provision of mulch displayed significantly the minimum curd yield (13.07 t ha^-1^).

**Table 3:** Interaction effects of different methods and levels of irrigation and mulches on broccoli curd yield.

Treatments	Curd yield (t ha^-1^)											
	2014–15			2015–16			2016–17			Pooled		
	M_0_	M_1_	M_2_	M_0_	M_1_	M_2_	M_0_	M_1_	M_2_	M_0_	M_1_	M_2_
I_1_	13.63	17.45	14.85	12.45	14.33	13.93	13.12	16.18	14.2	13.07	15.99	14.33
I_2_	14.48	18.31	17.22	14.33	16.2	15.88	14.33	16.86	16.3	14.38	17.12	16.47
I_3_	16.72	18.92	17.64	16.58	18.46	17.82	17.22	20.14	17.69	16.84	19.17	17.72
I_4_	16.85	19.04	17.72	16.72	18.53	17.94	17.57	20.46	17.8	17.05	19.34	17.82
SEm±	0.22			0.23			0.25			0.19		
LSD (*P≤* 0.05)	1.31			1.42			1.54			1.17		

I_1_: surface irrigation at IW/CPE 1.0, I_2_: drip irrigation at 0.6 ETc_,_ I_3_: drip irrigation at 0.8 ETc, I_4_: drip irrigation at 1.0 ETc; M_0_: nomulch, M_1_: black plastic mulch, M_2_: paddy straw mulch, ETc: crop evapotranspiration.

### 3.2. Seasonal actual crop evapotranspiration and water productivity

The depth of irrigation water applied averaging over the 3-year cropping period by drip irrigation at 0.6, 0.8 and 1.0 ETc was 71.00, 94.61 and 118.25 mm, respectively and 150 mm for surface irrigation (Table 4). The mean effective rainfall was 19.85 mm. The mean soil water contribution ranged between 34.23 mm and 36.09 mm. The estimated seasonal reference evapotranspiration (ET), crop evapotranspiration (ETc) and actual crop evapotranspiration (ETa) under different methods and levels of irrigation for broccoli at different growing periods are furnished in Fig 2. The estimated seasonal crop water uses or ETa was 146.94, 169.78 and 192.33 mm through drip irrigation at 0.6, 0.8 and 1.0 ETc, respectively and 225.29 mm for surface irrigation (Table 4).

**Table 4:** Water balance components, seasonal actual crop evapotranspiration (ETa), crop water productivity (CWP), irrigation water productivity (IWP) and water saving for broccoli under different levels and methods of irrigation and mulches (average data of 3-year).

Treat-ment	Profile water (mm)	Effective rainfall (mm)	Irrigation water (mm)	≠ETa (mm)	CWP (kg m^-3^)	IWP (kg m^-3^)	Water saving (%)	Extra land for irrigation (%)
**Irrigation (I)**								
I_1_	35.44	19.85	150.00	225.29	6.42	9.64	-	-
I_2_	36.09	19.85	71.00	146.94	10.88	22.52	52.67	53.32
I_3_	35.32	19.85	94.61	169.78	10.55	18.93	36.93	32.69
I_4_	34.23	19.85	118.25	192.33	9.40	15.28	21.17	17.14
**Mulch (M)**								
M_0_	34.15	19.85	108.46	182.46	8.40	14.13	-	-
M_1_	37.08	19.85	108.46	185.39	9.66	16.51	-	-
M_2_	34.57	19.85	108.46	182.88	9.07	15.29	-	-
**Interaction (I × M)**								
I_1_M_0_	34.15	19.85	150.00	224.00	5.83	8.71	-	-
I_1_M_1_	36.93	19.85	150.00	226.78	7.05	10.66	-	-
I_1_M_2_	35.23	19.85	150.00	225.08	6.37	9.55	-	-
I_2_M_0_	34.92	19.85	71.00	145.77	9.86	20.25	-	-
I_2_M_1_	38.12	19.85	71.00	148.97	11.49	24.11	-	-
I_2_M_2_	35.22	19.85	71.00	146.07	11.28	23.20	-	-
I_3_M_0_	34.35	19.85	94.61	168.81	9.98	17.80	-	-
I_3_M_1_	37.05	19.85	94.61	171.51	11.18	20.26	-	-
I_3_M_2_	34.55	19.85	94.61	169.01	10.48	18.73	-	-
I_4_M_0_	33.18	19.85	118.25	191.28	8.91	14.42	-	-
I_4_M_1_	36.22	19.85	118.25	194.32	9.95	16.36	-	-
I_4_M_2_	33.29	19.85	118.25	191.39	9.31	15.07	-	-

I_1_: surface irrigation at IW/CPE 1.0, I_2_: drip irrigation at 0.6 ETc_,_ I_3_: drip irrigation at 0.8 ETc, I_4_: drip irrigation at 1.0 ETc; M_0_: no mulch, M_1_: black plastic mulch, M_2_: paddy straw mulch; ≠ Common irrigation of 20 mm depth for uniform plant establishment and irrigation regime maintenance.

Mean maximum CWP and IWP (10.88 and 22.52 kg m^-3^, respectively) was obtained with the higher deficit drip irrigation at ETc 0.6 followed by moderate deficit drip irrigation at 0.8 ETc (10.55 and 18.93 kg m^-3^, respectively) and optimal drip irrigation at 1.0 ETc (9.40 and 15.28 kg m^-3^, respectively), whereas surface irrigation accomplished mean minimum CWP and IWP (6.42 and 9.64 kg m^-3^, respectively). The overall increase in CWP and IWP was 69.5 and 133.6%, 64.3 and 96.4% and 46.4 and 58.5% for drip irrigation scheduling at 0.6, 0.8 and 1.0 ETc, respectively over the corresponding values in surface irrigation.

Regardless of methods and levels of irrigation, BPM was found to be more effective in displaying higher CWP and IWP (9.66 and 16.51 kg m^-3^, respectively) than PSM (9.07 and 15.29 kg m^-3^, respectively), while without mulched or open soil condition recorded the lower CWP and IWP (8.40 and 14.13 kg m^-3^, respectively). The mean increase in CWP and IWP was 15.0 and 16.8%, respectively for BPM and 8.0 and 8.2%, respectively for PSM over the related values in without mulched treatment.

The interaction effects between irrigation and mulching showed that moderate drip irrigation level at 0.6ETc complemented with BPM recorded the highest CWP (11.49 kg m^-3^) and IWP (24.11 kg m^-3^) than the other treatment combinations. The treatment combination of surface irrigation without mulch, on the contrary, demonstrated the lowest CWP (5.83 kg m^-3^) and IWP (8.71 kg m^-3^).

### 3.3. Land and water utilization

The data on water saving and additional land area likely to be irrigated due to saving of water through the imposition of drip irrigation system is presented in Table 4. The results indicated that the higher level of deficit drip irrigation at 0.6 ETc saved about 52.67% of water as compared to the surface irrigation, which could bring about 53.32% of additional land area under drip irrigated crop. Similarly, the moderate level of deficit drip irrigation at 0.8 ETc could save as much as 36.93% of water in comparison with surface irrigation. On utilizing this saved water, it was possible to irrigate an additional land area of 32.69%. Likewise, the optimum level of drip irrigation at 1.0 ETc was found to save about 21.17% of water as compared to surface irrigation which could bring an extra land area of 17.14% under drip irrigation.

### 3.4. Water-yield production function

A strong second-degree polynomial relationship was best fitted between Y versus I and Y versus ET_a_ (Fig 3). The predicted regression equations are given below:

$$Y=-0.0019I^{2}+0.4073I-3.2607{\;(R}^{2}=0.9968)$$ (8)

$$Y=-0.0019{{ET}_{a}}^{2}+0.7031{ET}_{a}-45.471{\;(R}^{2}=0.9987)$$ (9)

The coefficient of determination (R^2^) was found to be 0.99768 for Eq 7 and 0.9987 for Eq 8 which were statistically highly significant. Mean maximum broccoli curd yield (18.25 t ha^-1^) was estimated at the inflection point of the quadratic regression curve with 105 mm of irrigation water (I) and 180 mm of ETa. The projected yield reductions as affected by the adopted irrigation treatment for surface irrigation, drip irrigation at 0.6 ETc, 0.8 ETc and 1.0 ETc were 20.8, 12.4, 1.9 and 1.0%, respectively.

### 3.5. Crop yield response factor

**Fig 4:**
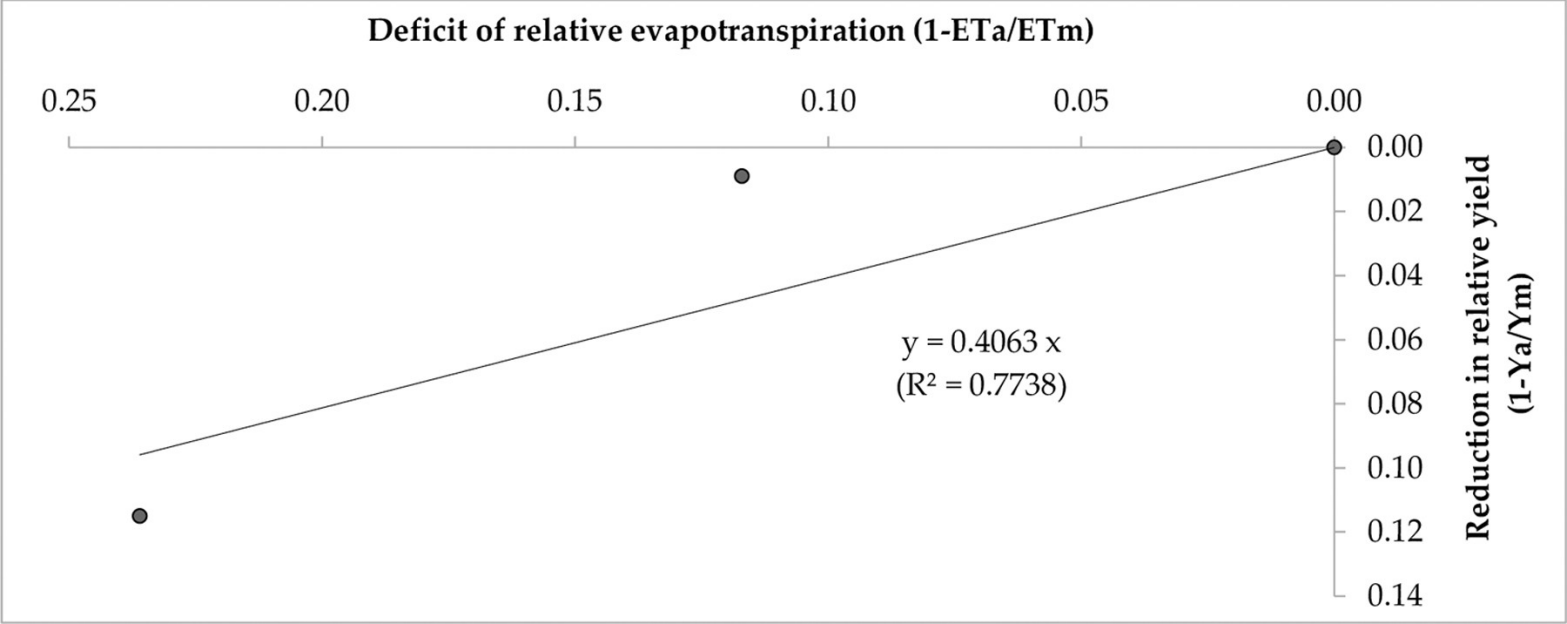
Relationship between relative yield deficit and relative evapotranspiration deficit (average of 3 cropping seasons).

Broccoli yield response to water stress was obtained from the linear relationship between the seasonal relative yield deficits in response to the relative actual crop evapotranspiration deficits (Fig 4).

It shows the response of curd yield with a concomitant decrease in water consumption. In other words, it explains the decline in yield with respect to per unit decrease in water use. The slope of the curve denotes the seasonal crop yield response factor (Ky) for the whole growing season and was estimated to be 0.41 with a high coefficient of determination (R^2^ = 0.77). The values of Ky consistently increased with increasing water deficit (Table 5).

**Table 5:** Relationship between decrease in relative water use and decrease in relative yield for drip irrigated broccoli (average data of 3-year).

Drip irrigation	ETa (mm)	ETm (mm)	(1-ETa/ETm)	Ya (t ha^-1^)	Ym (tha^-1^)	(1-Ya/Ym)	Ky
0.6 ETc	146.94	192.33	0.236	15.99	18.07	0.115	0.488
0.8 ETc	169.78	192.33	0.117	17.91	18.07	0.009	0.076
1.0 ETc	192.33	192.33	0	18.07	18.07	0	0

### 3.6. Economic analysis

The detailed overall total cost of cultivation including the fixed cost for surface irrigation and gravity-fed drip irrigation; operational cost for irrigation, mulch materials and other inputs used; and gross return, net return and benefit-cost ratio (BCR under) different irrigation and mulch treatments on broccoli is summarized in Table 6. The average cost of cultivation irrespective of mulch materials was found maximum in drip irrigation at 1.0 ETc followed by surface irrigation, drip irrigation at 0.8 ETc and 0.6 ETc, respectively. The results showed that average higher gross return (₹201320 ha^-1^), maximum net return (₹161937 ha^-1^) and BCR (4.11) were accomplished with moderate deficit drip irrigation at 0.8 ETc accommodated with BPM (I_3_M_1_) which were followed immediately by moderate deficit drip irrigation at 0.8 ETc with PSM (I_3_M_2_) and moderate deficit drip irrigation at 0.8 ETc without mulch (I_3_M_0_) with the corresponding value of ₹186025 ha^-1^, ₹148142 ha^-1^ and 3.91 and ₹176820 ha^-1^, ₹140437 ha^-1^ and 3.86, respectively. Optimum drip irrigation at 1.0 ETc with BPM (I_4_M_1_) recorded relatively higher gross return (₹203105 ha^-1^), net return (₹160012 ha^-1^) and moderate BCR (3.71). On the other hand, the corresponding values were moderate in higher deficit drip irrigation at 0.6 ETc and were lower in surface irrigation with and without mulching conditions.

**Table 6:** Economics of broccoli cultivation as influenced by different levels and methods of irrigation and mulches (average data of 3-year).

Treatments	Total cost of cultivation (₹ ha^-1^)	Curd yield (t ha^-1^)	Gross return (₹ ha^-1^)	Net return (₹ ha^-1^)	Benefit:cost ratio
I_1_M_0_	36966	13.07	137200	100234	2.71
I_1_M_1_	39966	15.99	167860	127894	3.20
I_1_M_2_	38466	14.33	150430	111964	2.91
I_2_M_0_	36272	14.38	150990	114718	3.16
I_2_M_1_	39272	17.12	179795	140523	3.58
I_2_M_2_	37772	16.47	172900	135128	3.58
I_3_M_0_	36383	16.84	176820	140437	3.86
I_3_M_1_	39383	19.17	201320	161937	4.11
I_3_M_2_	37883	17.72	186025	148142	3.91
I_4_M_0_	40093	17.05	178990	138897	3.46
I_4_M_1_	43093	19.34	203105	160012	3.71
I_4_M_2_	41593	17.82	187110	145517	3.50

I_1_: surface irrigation at IW/CPE 1.0, I_2_: drip irrigation at 0.6 ETc_,_ I_3_: drip irrigation at 0.8 ETc, I_4_: drip irrigation at 1.0 ETc; M_0_: no mulch, M_1_: black plastic mulch, M_2_: paddy straw mulch; Marketable price of broccoli curd @ ₹ 10500 t^-1^.

## 4. Discussion

### 4.1. Effect of irrigation and mulch on curd yield

The increased curd yield with drip irrigation at 1.0 ETc and 0.8 ETc over surface irrigation might be attributed to the enhanced water utilization as a result of better distribution of water in the active root zone of broccoli due to constant maintenance of soil water near to field capacity in low suction [36], increased availability and concomitant uptake of nutrients by plant throughout the growing period [37] and excellent soil water-air balance with plenty oxygen concentration in the rhizosphere [17]. All these contributory factors might have stimulated enhanced physiological processes, active cell division and photosynthetic activities of plants [13]. The dramatic decline in curd yield in drip irrigation at 0.6 ETc was mainly due to acute soil water stress-causing reverse osmosis in plants and was unable to replenish the crop water requirement [38, 39]. Surface irrigation, on the other hand, rendered extensive wetting zone as water spreading over the large area due to high hydraulic gradient causing higher soil evaporation, excessive losses of water and nutrients in deep percolation, chocking of soil aeration in first few days immediately after irrigation, higher soil water stress between two successive irrigation and in some critical growth period and reduced nutrient availability due to heavy irrigation load which ultimately proved to be detrimental for a drastic reduction of crop growth and yield [36]. The obtained results are in harmony with the findings of Pattanaik et al. [40] and Haris et al. [12] on okra; Singh et al. [41] on irrigated crops and Kashyap et al. [17] on broccoli.

Irrespective of methods and levels of irrigation, the increase in yield in mulched treatments over without mulched treatments could be due to better conservation, availability and consumption of soil water and nutrients by plants as a result of improved soil hydrothermal regimes and suppression of weed population [5, 40].

Higher yield with optimum (1.0 ETc) or moderately deficit (0.8 ETc) irrigation by gravity drip system supplemented with BPM was probably due to attainment of improved soil moisture status and soil temperature regimes in the rhizosphere and the less competition from minor weed infestation for water and nutrients in soil which facilitated more interception of incoming photosynthetic radiation and more absorption of water and nutrients through proliferated shallow root system resulting in increased the turgidity of cells, quicker cell division and enlargement leading to increased crop growth and yield [4, 42, 43].

### 4.2. Seasonal actual crop evapotranspiration and water productivity

There was a little quantitative variation in average soil water contribution towards ETa at all irrigation regimes. This reveals the fact that the deficit irrigated treatments or, drier soil moisture regime extracted more soil water from deeper layers in the soil profile than optimum irrigated treatment or, wetter soil moisture regime. The differences could be attributed to the shallow root structure of the broccoli plant and the methods and levels of irrigation imposed. Irrigation requirement was found higher in optimal drip irrigation at 1.0 ETc than the remaining deficit drip irrigation treatments as more irrigation water was needed by crop for maintaining such level. The average seasonal irrigation requirement for crops at 1.0 ETc was 66.5% more over 0.6 ETc and 25.0% more over 0.8 ETc. The mean irrigation requirement for surface irrigation at IW/CPE 1.0 was much higher as compared to drip irrigation treatments because of the higher need for irrigation water in this schedule. Likewise, the marked differences in ETa among the irrigation treatments were mostly due to the variable amounts of irrigation water applied.

The overall CWP and IWP for broccoli were greatly influenced by the quantum of yield obtained in response to the variability of applied irrigation water, soil profile water extraction and effective rainfall. These parametric values progressively decreased with incremental irrigation water supply. The lesser CWC and IWP in surface irrigation could be ascribed to the depressed yield as the bulk of applied water was lost through soil evaporation and deep percolation. This was the reason why the potential yield increase was not commensurate with applied water. These attendant problems can be negotiated by drip irrigation system where the low volume of water with higher frequency was applied directly into the rooting zone at right time and checking of water losses in evaporation, runoff and deep percolation, thus enabling the crop to absorb water more efficiently for increasing yield and thus resulted into higher CWP and IWP. It was conspicuous that the highest CWP and IWP were found with a higher level of deficit drip irrigation than moderate deficit and optimum levels of drip irrigation. This implied full utilization of applied water towards proportional production in the former treatment than in the later treatments. The results are almost similar to those of Rajurkar et al. [10] who demonstrated higher water productivity and water saving in drip-irrigated treatments as compared to surface irrigated treatment.

Regardless of irrigation regimes, different mulch treatments had an additional impact in increasing CWP and IWP over without mulched treatment mainly by way of yield improvement as a result of the increasing availability of water and nutrients in soils. The effect was more prominent in BPM than in PSM treatments. Relatively higher yield and CWP and IWP as noticed in different drip irrigation scheduling in association with BPM are corroborated with the works of Maheria et al. [44] who reported better soil water conservation by plastic sheet mulching and irrigation application through a low-pressure drip irrigation system that resulted in a significant increase in crop yield and higher water productivity in cumin.

### 4.3. Land and water utilization

Deficit irrigation is a valuable water management strategy in water-scarce regions where substantial amounts of water resources is saved which can be fruitfully used for other purposes or irrigating the extra units of land. The results showed that application of optimum (1.0 ETc) and deficit irrigation (0.8 to 0.6 ETc) scheduling through drip irrigation system offered to save 21.2 to 52.7% irrigation water as compared with surface irrigation. This saved water could provide an excellent opportunity to irrigate an additional area of 17.1 to 53.3% under drip irrigated broccoli. Increasing deficit drip irrigation could save relatively higher amounts of water and bring more area under irrigation than optimum drip irrigation. Thus in limited to scarce water resources, deficit irrigation management strategy using low-cost gravity drip irrigation system could be a viable alternative to accommodate more unirrigated area under irrigation scheme with compromisation of some economic yield reduction.

### 4.4. Water-yield production function

In curves 4a and 4b, the initial linear effects indicate the increase in broccoli curd yield was proportional to the increment of ‘I’ and ETa, reach almost a plateau with mild variation in yield with increasing ‘I’ and ETa, then exhibit the quadratic effects which display marked decline in yield with further increase in ‘I’ and ETa where the maximum amount of applied water was not economically utilized for production purpose. Fereres and Soriano [45] also noticed such curvilinear relations in water excess conditions when all the applied water are not used in crop ET and some fraction is unnecessarily lost.

The highest reduction in curd yield was observed with surface irrigation with a higher load of water application (50 mm depth each at 19–22 days interval) which was particularly due to the cyclic occurrence of water excess followed by water deficit in different magnitudes across the growth stages of plants. Likewise, a higher level of deficit drip irrigation at 0.6 ETc might have resulted in a more negative effect on plants through higher soil water stress at all the growth stages. Even the optimum drip irrigation at 1.0 ETc using a relatively higher amount of water application (118.25 mm) had a marginal effect on plants in discouraging yield. Thus the estimated 105 mm of irrigation water or 180 mm of ETa was found to be the optimal irrigation or ETa requirement, respectively for deriving maximum green curd yield of broccoli using the gravity drip irrigation system. The curvilinear model of relative yield with irrigation or ETa can successfully be employed in the Indo-Gangetic plains for projecting broccoli yield potential in relation to different irrigation water supply and rainfall conditions. The obtained results are in line with the findings of Kumar et al. [9] who recorded the significant polynomial relationships between bulb yield and applied irrigation water and crop evapotranspiration (ETc) under micro-sprinkler irrigated onion crop in the semi-arid climate of India. Bozkurt et al. [46] also found significant second-degree polynomial relationships between grain yield and irrigation water use on hybrid corn under the Mediterranean climatic conditions. Yazar et al. [47] observed a significant second-degree polynomial relationship between grain yield and seasonal water use (ET) in the Mediterranean climate of Southern Turkey. Conversely, Greaves and Wang [22] noticed significant linear relationships between grain yield and seasonal water applied and seasonal ETc for maize in southern Taiwan.

### 4.5. Crop yield response factor

Crop yield response factor (Ky) is the indicator of crop yield decrease with concomitant ETa deficit. Higher Ky value obtained under moderately to higher water stress due to imposition of drip irrigation level at 0.8 ETc and 0.6 ETc, respectively indicates that the plant will experience moderate to acute water stress and have greater yield losses when optimal water requirements for crops are not adjusted immediately. This also implies a high adverse impact of soil-water stress treatment on the curd yield. The observed seasonal Ky value of 0.41 explained a substantial yield loss of 0.41 t (410 kg) per mm of ETa deficit. On the other hand, Ayas et al. [11] reported a high Ky value of 1.04 for broccoli. Doorenbos and Kassam [24] suggested that if Ky values are above 1.0, the crop is sensitive to water stress and yield decrease is proportionally greater with an increase in water deficit, whereas Ky values below 1.0 indicate the crop is tolerant to water stress in some extents and yield decrease is proportionally lesser with increasing water deficit. This reveals the fact that a deficit irrigation management strategy with a gravity drip irrigation system in some growth stages of broccoli plants can be adopted on a larger scale in this agro-climatic region.

### 4.6. Economic analysis

The average cost of cultivation for different treatment combinations increased progressively according to the increase in cost towards increased investment on irrigation water and mulches. In each irrigation level, the incorporation of BPM substantially increased the cost of production than PSM. The gross return, net return and BCR for drip-irrigated broccoli depended upon the proportionate increase in green curd yield due to increase in irrigation water through drip system and the extra cost of production incurred towards system commission, irrigation charges and type of mulching material used. The corresponding values were relatively lower in surface irrigated crops complemented with different mulch materials. The higher monetary returns and BCR in mild deficit irrigation regime at 0.8 ETc by drip system coupling with BPM or PSM was probably due to the increase in yield on account of higher utilization of water and nutrients by elongated and proliferated root system as influenced by favourable soil hydrothermal regime in crop’s root zone (Table 6).

## 5. Conclusion

The results of the present study indicated that imposition of drip irrigation in broccoli with IW/CPE at 0.8 with black polythene mulch can be recommended to this locality considering its higher curd yield, CWP and economic aspects of crop production. Under the water-scarce condition, drip irrigation at 0.6 ETc with black polythene mulch can be an alternative for broccoli cultivation. Drip irrigation could save 21.2–52.7% water over surface irrigation which accommodated 17.1 to 53.3% additional area under irrigation. Yield response factor and water-yield production function suggested the potential yield decrease in relation to increased deficit irrigation. However, a deficit drip irrigation scheduling with 0.8 ETc at a 3-day interval is optimum for increased curd yield, water productivity and economics of broccoli.
